# High fat diet sensitizes fibromyalgia-like pain behaviors in mice via tumor necrosis factor alpha

**DOI:** 10.1371/journal.pone.0190861

**Published:** 2018-02-14

**Authors:** Dan Tian, Miao Tian, Leilei Zhang, Peng Zhao, Yunfeng Cui, Jinlong Li

**Affiliations:** 1 Department of Anesthesiology, Second hospital of Jilin University, Changchun, China; 2 Department of Gynecology, Second hospital of Jilin University, Changchun, China; 3 Department of Gastrointestinal Surgery, Second hospital of Jilin University, Changchun, China; University of South Alabama Mitchell Cancer Institute, UNITED STATES

## Abstract

Fibromyalgia (FM) and obesity are closely related. However, little is known about how obesity contributes to FM. Importantly, adequate evidence has shown that tumor necrosis factor alpha (TNF-α) plays a critical role in obesity. Thus, we hypothesized that obesity-induced TNF-α release may potentiate FM-associated pain. To test this hypothesis, we investigated the role of TNF-α in the development of FM-like pain in a mouse model of acid saline injection-induced FM. Consistent with previous reports, we showed that repeated acid saline injections induced bilateral mechanical hyperalgesia, and this effect lasted for at least 4 days after acid saline injections. This phenomenon was associated with increased levels of TNF-α in plasma, muscles, and spinal cord. Furthermore, we found that 24 weeks of high fat diet treatment significantly potentiated acid saline-induced bilateral mechanical hyperalgesia. High fat diet-treated mice exhibited robustly increased levels of TNF-α in plasma, muscles, and spinal cord after acid saline injections compared with low fat diet-treated mice. Additionally, using immunofluorescence staining, we found that the number of TNF-α positive cells in dorsal root ganglion (DRG) was increased after acid saline injections, and high fat diet treatment further sensitized this increase. Finally, we reported that acid saline-induced FM-like pain behaviors were abolished in TNFRp55-/- mice, confirming the critical role of TNF-α in the development of FM-like pain. Taken together, our results suggested that high fat diet treatment may sensitize acid saline-induced FM-like pain via increasing TNF-α levels in plasma, muscles, and DRG.

## Introduction

Fibromyalgia (FM) is a complex syndrome characterized by widespread chronic musculoskeletal pain which is accompanied by fatigue, sleep, memory and mood issues. FM may affect up to 8% of population worldwide [1, 2]. While the underlying disease mechanisms are still unclear, FM associated pain may be contributed by multiple factors, including abnormal regulation of central pain modulation [3], dysregulated hypothalamic-pituitary-adrenal axis (HPA) [4], and immunological vulnerability [5].

Remarkably, these aforementioned factors are often seen in obesity. Specifically, obese people typically exhibit abnormalities in the regulation of the HPA axis [6, 7]. Obese humans [8–12] and rodents [13, 14] exhibit higher sensitivity to experimentally induced nociceptive stimuli. Obese people often report to have chronic pain disorders [15–17]. Numerous studies have suggested that FM and obesity are closely related. Studies showed that up to 50% of FM patients are obese and additional 28% are overweight [18–20]. In addition, FM patients have higher body mass index (BMI) than pain-free individuals [21, 22]. These results suggested that obesity or overweight is related to greater pain/tender sensitivity in FM [18–20]. However, little is known about how obesity contributes to FM.

Recently, studies have reported increased levels of proinflammatory cytokine tumor necrosis factor alpha (TNF-α), in the serum and biopsies of FM patients [23, 24], which might be associated with disease symptoms [25–27]. Intramuscular injection of TNF-α induces muscle hyperalgesia in rats [28]. Importantly, adequate evidence has shown that TNF-α plays a critical role in obesity [29–31]. Increased levels of TNF-α in plasma and muscles were seen in obese patients and rodents [32, 33]. Thus, it is likely that obesity-induced TNF-α release may potentiate FM associated pain. To test this hypothesis in the present study, we examined the effects of high fat diet treatment on FM-like pain in a mouse model of FM. We then further assessed the role of TNF-α in the development of FM.

## Materials and methods

### Animals

Female TNFRp55-/- (TNFR1 KO) mice were obtained from The Jackson Laboratory (ME USA). Female C57BL/6J mice were obtained from Shanghai Laboratory Animal Center (Shanghai, China). All experiments were started when female mice were 7–8 weeks old. Mice were housed in their home cages with a 12:12 light cycle with 21.0°C-23.0°C and 50%-60% humidity, and were given food and water *ad libitum*. All animal experiments were approved by the Institutional Animal Care and Use Committee of Second Hospital of Jilin University. The housing and treatment of the rats followed the guidelines of the “Guide for the Care and Use of Laboratory Rats” (Institute of Laboratory Animal Resources, Commission on Life Sciences 2011).

### High fat diet treatment

Experiments were performed on C57BL/6J mice. After arrival in the animal facility, mice initially received a low-fat diet (LFD) containing 10% fat (D12450B, Research Diets Inc., NJ, USA) for 1week until the experiment was initiated. Mice were randomly divided into control receiving a low-fat diet (LFD) and experimental group receiving a high-fat diet (HFD) containing 45% fat (D12451, Research Diets Inc., NJ, USA). Mice were then fed for 24 weeks. This treatment duration was decided based on literature [34–36]. Weight and blood glucose measurements (glucose diagnostic reagents; Sigma, St. Louis, MO, USA) were collected at the beginning of high fat diet treatment and at 24 weeks (**Table 1**). Mice were fasted for 3 hours prior to blood collection from the tail.

**Table 1:** Body weights, one-day food intake amounts, and blood glucose concentrations.

Weeks post-HFD		Low Fat Diet Group	High Fat Diet Group
24	Body weight (g)	38.4±2.2	58.7±1.9*
	Food intake (g)	3.6±0.3	7.1±0.3*
	Blood glucose (mmol/l)	7.2±0.3	9.0±0.5*

Data are expressed as Mean ± SEM, *n* = 8 per group. HFD: high fat diet. *Asterisks* represent the significant difference, relative to Low fat diet group. *p*< 0.05.

### Induction of Fibromyalgia (FM)

Following the high fat diet treatment, a mouse model of fibromyalgia was induced and adapted based on the methods described in literature [37]. Acid saline (20μL, pH 4.0) was injected into gastrocnemius muscle when mice were anesthetized with isoflurane (1%). Acid saline injection was repeated once at day 5 after the first injection to induce FM in mice. Control mice received saline injections under the same administration schedule. After treatment, mice were subjected to behavioral tests on the next day for 4 consecutive days.

### Analysis of TNF-α in plasma, muscles, and spinal cord

Mice were sacrificed after the last behavioral test, and samples of gastrocnemius muscles and spinal cord were collected. The tissue samples were then homogenized in 500 μl of buffer solution with protease inhibitors. The concentrations of TNF-α in plasma, muscles, and spinal cord were determined by enzyme linked immunosorbent assay (ELISA), using the Mouse TNF alpha ELISA Kit (ab100747, Abcam, Shanghai, China), based on the protocol recommended by the manufacturer. The limit of detection for TNF-α was 0.1 pg/mL. Experiment was repeated three times.

### Immunofluorescence staining

Mice were perfused transcardially with 4% paraformaldehyde under isoflurane anesthesia, and L3-L5 DRG tissues were collected. After being cryprotected with 30% sucrose, the tissues were then sectioned to a thickness of 15μm and were post-fixed with 4% paraformaldehyde. DRG sections were then incubated with blocking solution containing 0.1% Triton X-100, 3% BSA, and 0.02% sodium azide in phosphate-buffered saline for 2 h at room temperature, followed by incubation at with the anti-TNF-α antibody (1:300; ab1793, Abcam, Shanghai, China) prepared in blocking solution at 4°C overnight. The fluorescent-labeled secondary antibodies (Abcam, 1:200) specific to the IgG species were then used. All the fluorescent imaging was performed with the same laser power and exposure time by a CKX41 microscope with an Olympus U-RFLT50 Power Supply Unit (Olympus, Tokyo, Japan). The images were quantitatively analyzed by NIH ImageJ.

### Behavioral tests

Mechanical hyperalgesia was measured by testing the force of responses to stimulation with an electronic von Frey anesthesiometer (IITC, Shanghai, China). Briefly, mice were placed on a mesh floor 30 min before testing. Probes were perpendicularly applied to the central area of the hind paw with a gradual increase in pressure. After the withdrawal response, the intensity of the pressure was recorded automatically, and the results (an average of three measurements) were expressed as withdrawal threshold (g). The basal mechanical withdrawal threshold was 3.7 ± 0.1 g (mean ± SEM) before the beginning of experiment in TNFRp55-/- mice and C57BL/6J mice. There was no difference of basal mechanical withdrawal thresholds between groups in the same experiment.

### Statistical analyses

Data were analyzed using SPSS software (IBM, Inc., USA) and are presented as mean ± SEM. Data were analyzed by one-way analysis of variance (ANOVA) or multi-factorial ANOVA, followed by Bonferroni post hoc test, as appropriate. P<0.05 was considered to be significant.

## Results

### High fat diet treatment increased mechanical sensitivity in a mouse model of FM

**Fig 1:**
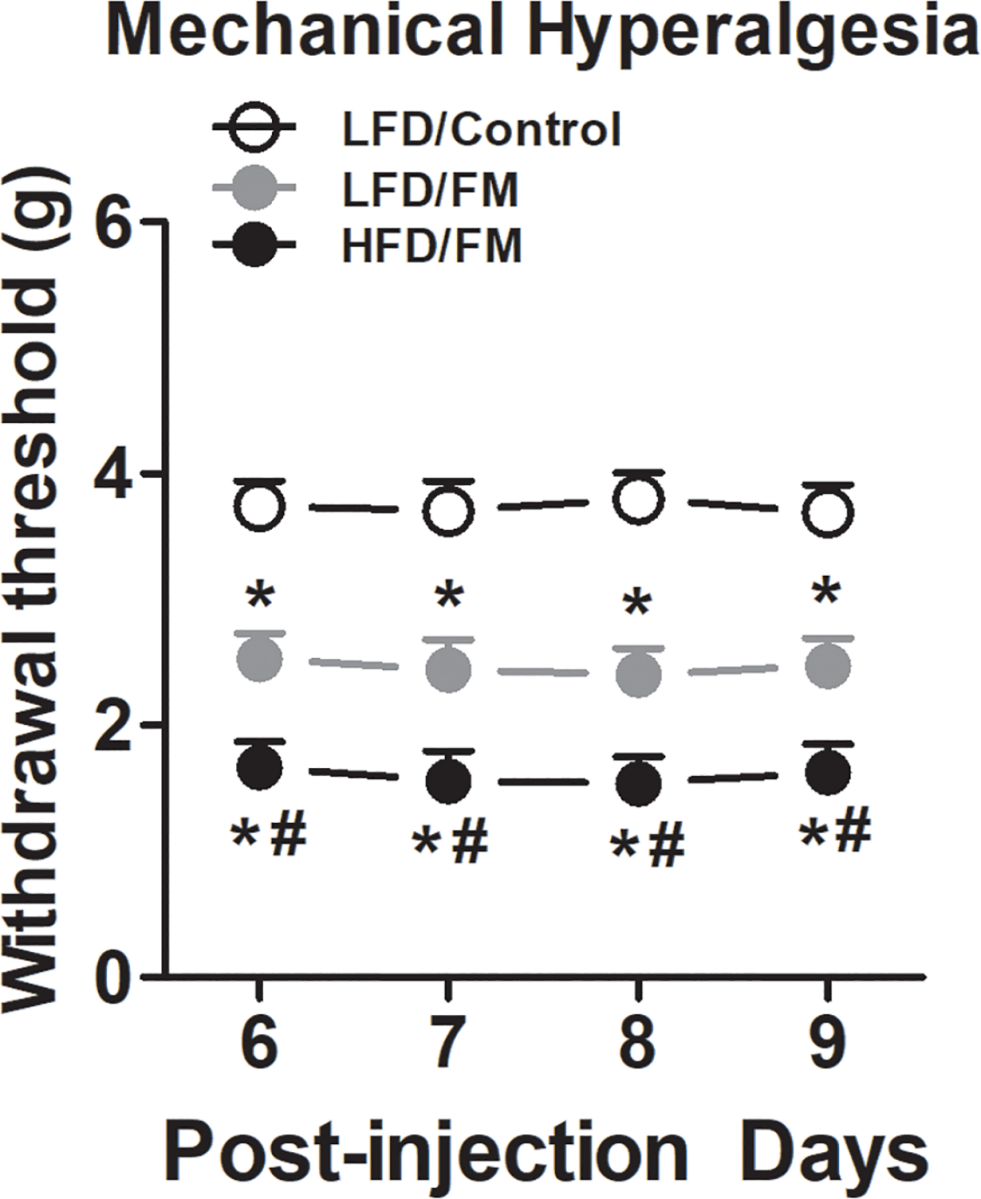
Effects of high fat diet treatment on acid saline injection-induced mechanical hypersensitivity. Following 24 weeks of high fat diet treatment, mice received repeated acid saline injections into the gastrocnemius muscle. Mechanical withdrawal thresholds were then measured using an electronic von Frey device for 4 consecutive days. Data are presented as mean ± SEM. Bonferroni correction for multiple comparisons. *represent the significant difference (p<0.05) to saline control mice. ^*#*^represent the significant difference (p<0.05) to low fat diet mice. N = 8/group.

Consistent with previous reports [37–39], normal saline injections in mice (i.e., control group) did not induce ipsilateral mechanical hyperalgesia (Fig 1, n = 8). Intramuscular injections of an acidic saline (pH = 4.0) into the gastrocnemius muscle of mice induced bilateral mechanical hyperalgesia (Fig 1, n = 8). These effects lasted for at least 4 days (i.e., day 6–9). Furthermore, previous high fat diet treatment for 24 weeks significantly enhanced mechanical hyperalgesia (Fig 1, n = 8).

### High fat diet treatment increased the levels of TNF-α in plasma cord and muscles in a mouse model of FM

**Fig 2:**
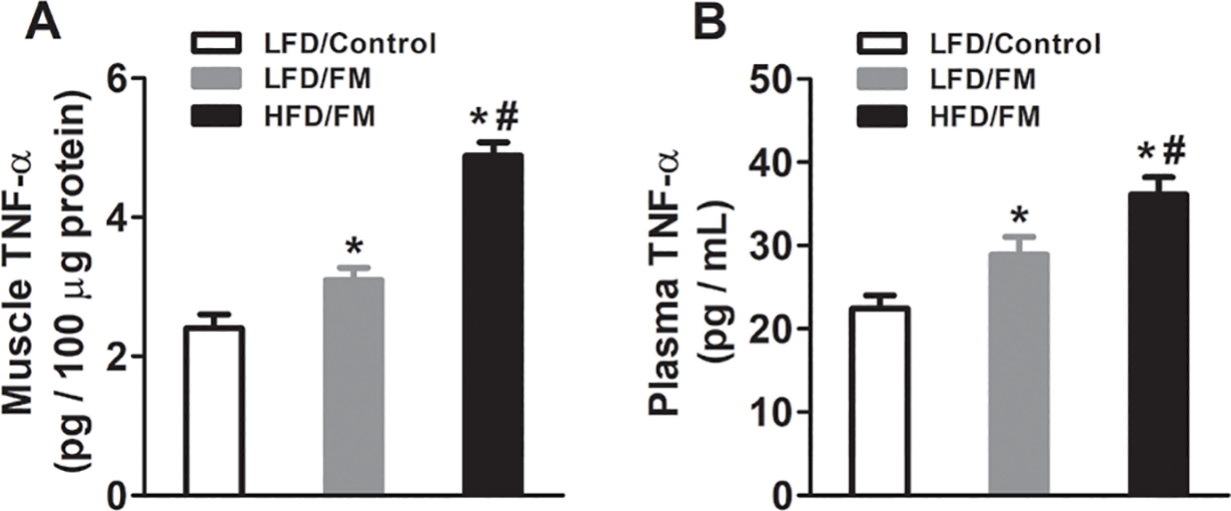
Levels of TNF-α in muscles and plasma of mice. Following 24 weeks of high fat diet treatment, mice received repeated acid saline injections into the gastrocnemius muscle. Samples of plasma and gastrocnemius muscles were collected. The concentrations of TNF-α in (**A**) muscles and (**B**) plasma were determined by ELISA. The limit of detection for TNF-α was 0.1 pg/mL. *represent the significant difference (p<0.05) to saline control mice. ^*#*^represent the significant difference (p<0.05) to low fat diet mice. N = 8/group.

To determine whether high fat diet altered the levels of TNF-α in plasma and muscles, we collected blood and muscle tissues after the last behavioral test. We found that intramuscular injections of an acidic saline (pH = 4.0) into the gastrocnemius muscle of mice increased the levels of TNF-α in both plasma and muscles (Fig 2A and 2B, n = 8) compared with normal saline control mice. Furthermore, previous high fat diet treatment for 24 weeks significantly potentiated the levels of TNF-α in both plasma and muscles (Fig 2A and 2B, n = 8).

### High fat diet treatment increased the levels of TNF-α in spinal cord and DRG in a mouse model of FM

**Fig 3:**
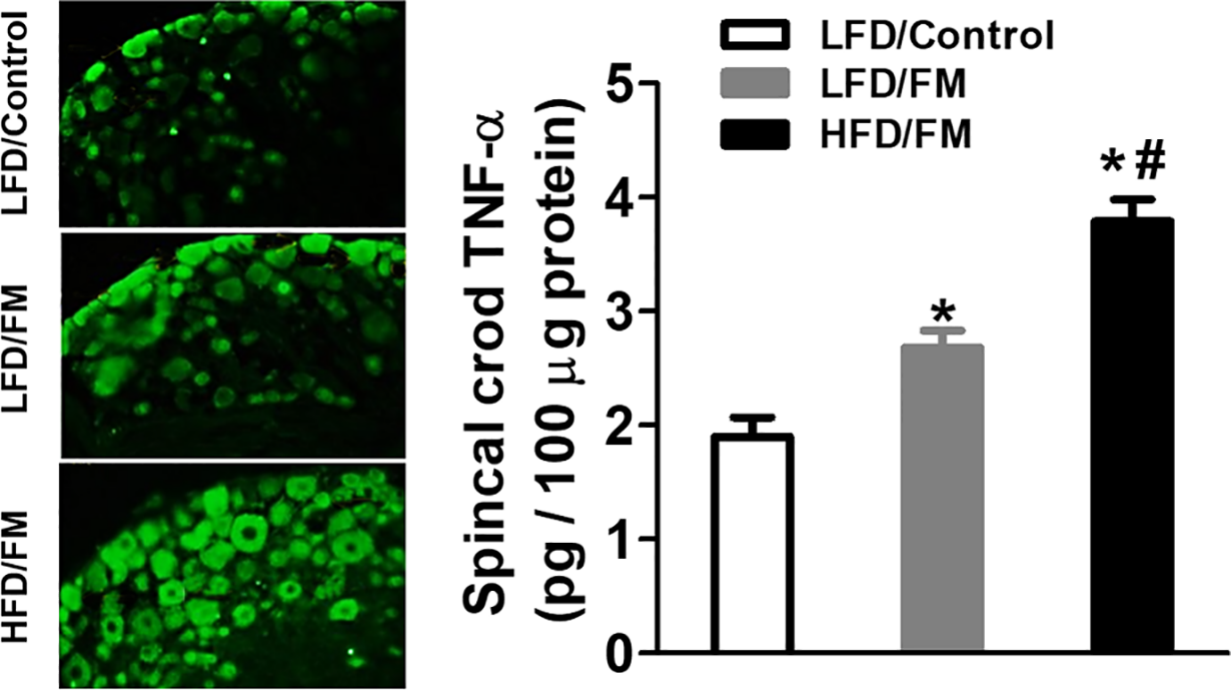
Levels of TNF-α in spinal cord and DRG. Mice were sacrificed after the last behavioral test, and samples of spinal cord and L3-L5 DRG tissues were collected in separate groups of mice. The concentrations of TNF-α in spinal cord were determined by ELISA. L3-L5 DRG tissues were sectioned to a thickness of 15μm and underwent immunofluorescent staining. The images were quantitatively analyzed by NIH ImageJ. *represent the significant difference (p<0.05) to saline control mice. ^*#*^represents the significant difference (p<0.05) to low fat diet mice. N = 8/group.

To determine whether high fat diet altered the levels of TNF-α in spinal cord and DRG, we collected spinal cord and L3-L5 DRG tissues after the last behavioral test. We found that intramuscular injections of an acidic saline (pH = 4.0) into the gastrocnemius muscle of mice increased the levels of TNF-α in spinal cord (Fig 3, n = 8) compared with normal saline control mice. Furthermore, previous high fat diet treatment for 24 weeks significantly potentiated the levels of TNF-α in spinal cord (Fig 3, n = 8). Additionally, we conducted immunofluorescence staining to assess the levels of TNF-α in the DRG. Similarly, immunofluorescent labeling visualized by green fluorescence indicated that TNF-α levels in the DRG were increased 4 days after FM induction (Fig 3, n = 8), and were further potentiated by previous high-fat diet treatment (Fig 3, n = 8).

### Acid saline injections failed to induce FM-like pain behavior in TNFRp55-/- mice

**Fig 4:**
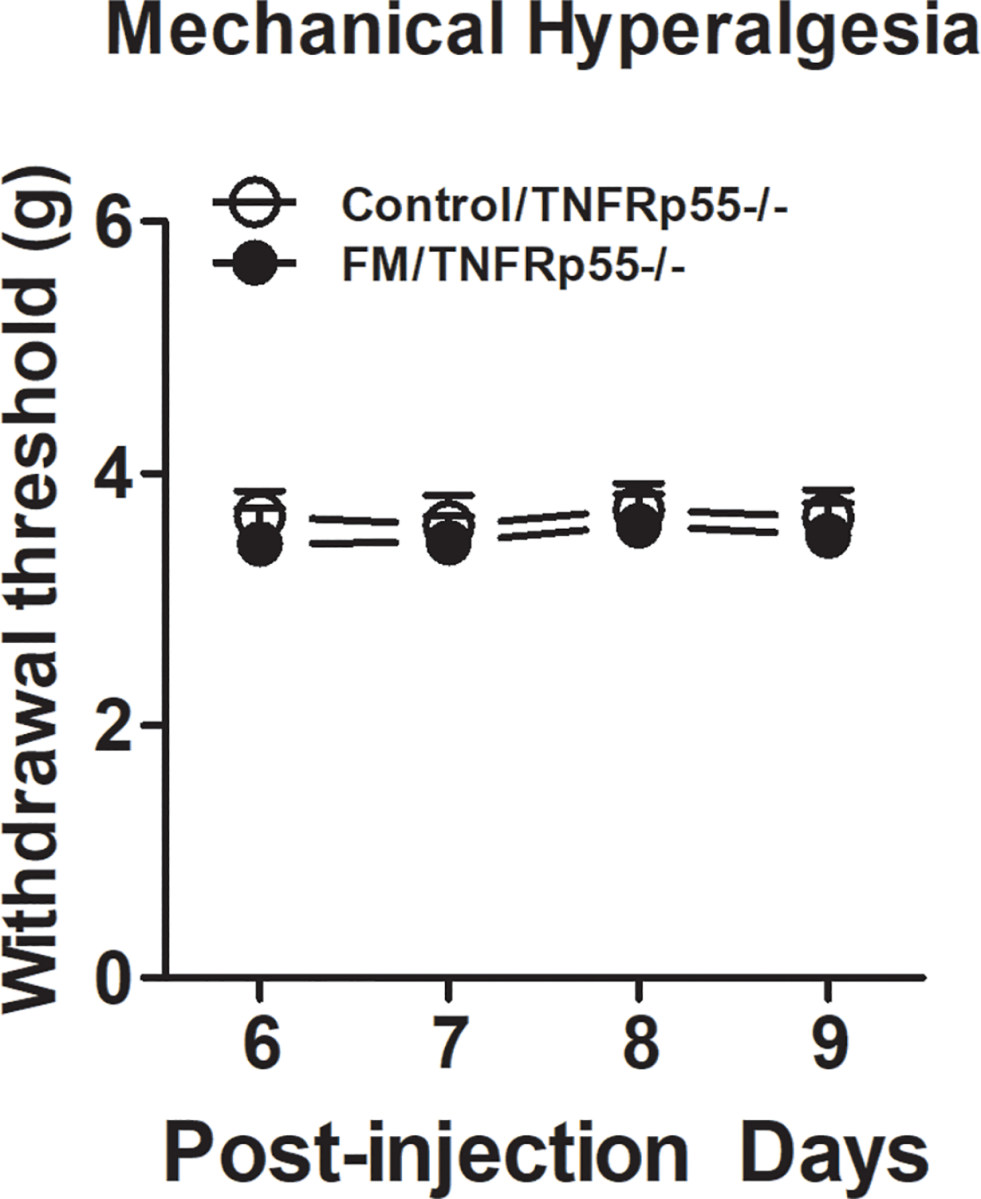
Effects of high fat diet treatment on acid saline injection-induced mechanical hypersensitivity in female TNFRp55-/- mice. Following 24 weeks of high fat diet treatment, mice received repeated acid saline injections into the gastrocnemius muscle. Mechanical withdrawal thresholds were then measure using an electronic von Frey device for 4 consecutive days. Data are presented as mean ± SEM. N = 8/group.

To explore the putative mechanisms underlying the role of TNF-α in the development of FM, we examined the mechanical hyperalgesia in TNFRp55-/- mice after intramuscular injections of an acidic saline (pH = 4.0). We found that intramuscular injections of an acidic saline (pH = 4.0) into the gastrocnemius muscle of mice did not induce bilateral mechanical hyperalgesia (Fig 4, n = 8).

## Discussion

In the present study, we investigated the role of TNF-α in the development of FM-like pain in a mouse model of FM. Based on published literature, we first established FM-like pain behaviors in mice using acid saline injections. Consistent with previous reports, we showed that repeated acid saline injections induced bilateral mechanical hyperalgesia, and this effect lasted for at least 4 days after acid saline injections. This phenomenon was associated with increased levels of TNF-α in plasma, muscles, and spinal cord, suggesting TNF-α may contribute to the widespread FM-like pain behaviors. Using this mouse model of FM-like pain, we further evaluated the effects of high fat diet treatments on the status of FM-like pain. We found that 24 weeks of high fat diet treatment significantly potentiated acid saline-induced bilateral mechanical hyperalgesia. Furthermore, we found that high fat diet-treated mice exhibited robustly increased levels of TNF-α in plasma, muscles, and spinal cord after acid saline injections compared with low fat diet-treated mice. Additionally, using immunofluorescence staining, we found that the number of TNF-α positive cells in DRG was increased after acid saline injections, and high fat diet treatment further sensitized this increase. Finally, we reported that acid saline-induced FM-like pain behaviors were abolished in TNFRp55-/- mice, confirming the critical role of TNF-α in the development of FM-like pain. Taken together, our results suggested that high fat diet treatment may sensitize acid saline-induced FM-like pain via increasing TNF-α levels in plasma, muscles, and DRG.

To study the disease mechanisms of FM, various rodent models have been established [40, 41]. For instance, previous studies have shown that injections of acidic saline into the muscles of mice can reliably induce pain symptoms similar to those of FM patients with minimal histopathological changes [40]. This FM mouse model exhibits long-lasting and widespread mechanical hyperalgesia without muscle damage, fatigue, sympathetic predominance or altered central sensitization [42–44]. However, unlike animal models for other types of nociceptive and neuropathic pain, it is relatively difficult to mimic FM etiologies in animal models because it is still unknow about its etiology. Therefore, the acid saline injection model of FM-like pain only mimics the symptomology and management profile of the disease rather than the mechanisms [40]. Nonetheless, this model mimics one important feature in FM that is the lack of tissue injury [40]. Furthermore, there is a high correlation between pain and co-morbidities, including fatigue, depression, and anxiety in FM. Thus, ideal animal models of FM should also simulate the development of these symptoms. However, no studies have reported that depression and anxiety-like behaviors were seen in acid saline injection model of FM [40]. In fact, other FM models such as biogenic amine depletion and sound stress models have been used to simulate depression and anxiety-like behaviors in rodents [40, 41]. Therefore, future studies will be important to validate our findings in multiple animal model of FM.

Our study focused on female mice, because clinical evidence shows that FM predominantly occurs in women [45–47]. However, obesity is similarly prevalent in males and females [48]. Importantly, animal studies have indicated that TNF-α production in obese male and female may be substantially different. For instance, there is a general lack of TNF-α autoamplification in obese female mice [49], suggesting that obese male mice may even have a higher response in TNF-α production after acid saline injection. While clinical evidence and previous animal studies all suggest that studies need to be performed in both male and female animals, because sex differences are likely to be important in the development and maintenance of FM-like symptoms. However, few animal models have been tested in both males and females. Thus, it will be necessary to test the effects of high fat diet on the development of FM-like pain in male mice in the future.

Previous studies indicated that FM-like syndrome may result from activation of vanilloid type 1 receptors (TRPV1s) or acid sensing ion channels (ASICs), which are voltage-insensitive cationic channels gated by extracellular protons [50]. Importantly, pH levels are often reduced in peripheral tissues of FM patients [51]. Furthermore, it has been shown that protons can activate the terminals of nociceptors. Electrophysiology recordings of DRG neurons have demonstrated that acid can activate TRPV1, leading to an up-regulation of CGRP expression in DRG via CaMK-CREB cascade, a series of events that may be associated with inflammatory pain [52]. As decreases in pH are associated with inflammation, it is likely that inflammatory mediators, such as TNFα, could sensitize the pH response in muscles or DRG. In fact, TRPV1 shows an increase in expression after prior treatment with TNF-α in cultured fibroblast-like synoviocytes [53], suggesting a direct interaction of TNF-α and TRPV1. Interestingly, in contrast to TRPV1, application of TNF-α failed to alter the mRNA levels of ASIC1, ASIC2 and ASIC3 in the DRG, suggesting TNF-α may not modulate expression of ASICs in DRG [54, 55]. However, similar to TRPV1, TNF-α mediated inflammation may reduce the pH and trigger the activation of ASICs leading to enhanced muscle pain. Thus, given that our results clearly showed an increase in TNF-α level in DRG, future experiments should investigate an interaction between inflammatory mediators and the pH response in DRG neurons.

Adding to existing literature, our study first reported that TNFRp55-/- mice is resistant to the development of FM-like pain behaviors after repeated acid saline injections. In fact, previous studies have demonstrated that multiple molecules are involved in FM-like pain. Besides ASICs and TRPV1, these molecules including N-methyl-D-aspartate receptors (NMDARs), voltage-gated calcium channels and voltage-activated sodium channels, as well as substance P have been implicated in mouse models of FM pain [37, 42, 56–58]. Importantly, TNF-α has been shown to interact with these molecules. Specifically, TNF-α can increase NMDAR function in spinal cord neurons [59]. In nociceptive DRG neurons, TNF-α decreases voltage-gated calcium channel currents, and increases voltage-activated sodium channels currents [60]. Furthermore, substance P release is enhanced in DRG cultures after chronic TNF-α treatment [61]. These results together suggest that TNF-α may play a central role in mouse models of FM pain.

In summary, our study was the first to report the role of TNF-α in the development of FM-like pain. Consistent with a lot of previous studies that inhibition of TNF-α-mediate neuroinflammation signaling can improve pain-like behavior in various mouse models of pain, these findings together suggested that TNF-α may have overlapping mechanisms that contribute to attenuation of distinct types of pain. In fact, peripheral synaptic transmission from DRG neurons to the spinal cord dorsal horn is crucial for pain signaling [62, 63], which is then transferred into the brain for processing the pain sensation and responses [64]. In our study, we reported that obesity sensitized acid saline-induced TNF-α production in the DRG. Thus, it is likely that increased DRG TNF-α production in obesity may contribute to the maintenance of FM-like pain behaviors. Future studies will be also important to explore the effects of TNF-α inhibition on the development of acid saline-induced FM-like pain behaviors, and dissect the putative role of TNF-α subpopulations (muscle, plasma, or spinal cord) in modulations of this phenomenon. This line of research will not only be important in advancing our knowledge in the neuropathological mechanisms of FM, but may provide an effective pharmacotherapeutic intervention in treating FM patients.
